# CoCUN, a Novel Ubiquitin Binding Domain Identified in N4BP1

**DOI:** 10.3390/biom9070284

**Published:** 2019-07-17

**Authors:** Ridvan Nepravishta, Federica Ferrentino, Walter Mandaliti, Anna Mattioni, Janine Weber, Simona Polo, Luisa Castagnoli, Gianni Cesareni, Maurizio Paci, Elena Santonico

**Affiliations:** 1 School of Pharmacy East Anglia, University of Norwich, Norwich NR4 7TJ, UK; 2 Department of Biology, University of Tor Vergata, 00133 Rome, Italy; 3 Department of Chemical Sciences and Technologies, Tor Vergata University, 00133 Rome, Italy; 4 IFOM, The FIRC Institute for Molecular Oncology, 20139 Milan, Italy; 5 DIPO, Dipartimento di Oncologia ed Emato-oncologia, Università degli Studi di Milano, 20122 Milan, Italy; 6 Fondazione Santa Lucia Istituto di Ricovero e Cura a Carattere Scientifico (IRCCS), 00143 Rome, Italy

**Keywords:** ubiquitin-binding domains (UBDs), nuclear magnetic resonance spectroscopy, cullin binding domain associating with NEDD8 (CUBAN), KH And NYN domain containing (KHNYN), neural precursor cell expressed developmentally downregulated gene 8 (NEDD8)

## Abstract

Ubiquitin binding domains (UBDs) are modular elements that bind non-covalently to ubiquitin and act as downstream effectors and amplifiers of the ubiquitination signal. With few exceptions, UBDs recognize the hydrophobic path centered on Ile44, including residues Leu8, Ile44, His68, and Val70. A variety of different orientations, which can be attributed to specific contacts between each UBD and surface residues surrounding the hydrophobic patch, specify how each class of UBD specifically contacts ubiquitin. Here, we describe the structural model of a novel ubiquitin-binding domain that we identified in NEDD4 binding protein 1 (N4BP1). By performing protein sequence analysis, mutagenesis, and nuclear magnetic resonance (NMR) spectroscopy of the ^15^N isotopically labeled protein, we demonstrate that a Phe-Pro motif in N4BP1 recognizes the canonical hydrophobic patch of ubiquitin. This recognition mode resembles the molecular mechanism evolved in the coupling of ubiquitin conjugation to endoplasmic-reticulum (ER) degradation (CUE) domain family, where an invariant proline, usually following a phenylalanine, is required for ubiquitin binding. Interestingly, this novel UBD, which is not evolutionary related to CUE domains, shares a 40% identity and 47% similarity with cullin binding domain associating with NEDD8 (CUBAN), a protein module that also recognizes the ubiquitin-like NEDD8. Based on these features, we dubbed the region spanning the C-terminal 50 residues of N4BP1 the CoCUN domain, for *Cousin of CUBAN*. By performing circular dichroism and ^15^N NMR chemical shift perturbation of N4BP1 in complex with ubiquitin, we demonstrate that the CoCUN domain lacks the NEDD8 binding properties observed in CUBAN. We also show that, in addition to mediating the interaction with ubiquitin and ubiquitinated substrates, both CUBAN and CoCUN are poly-ubiquitinated in cells. The structural and the functional characterization of this novel UBD can contribute to a deeper understanding of the molecular mechanisms governing N4BP1 function, providing at the same time a valuable tool for clarifying how the discrimination between ubiquitin and the highly related NEDD8 is achieved.

## 1. Introduction

Ubiquitin (Ub) and ubiquitin-like (Ubl) proteins are small polypeptides that are covalently conjugated to various target proteins, leading to proteasome-dependent or independent degradation and changes in their localization and/or in their activity [1]. In order to correctly interpret these modifications, the cell requires Ub/Ubl adaptors called ubiquitin receptors, which contain one or more ubiquitin binding domains (UBDs) that transduce the post-translation modification in the appropriate cellular response [2,3,4]. The ubiquitin molecule consists of a globular core called the β-grasp fold, comprising five-stranded mixed β-sheet and an α-helix, from which a flexible carboxyl-terminal tail protrudes. Most of the ubiquitin binding domains identified to date recognize a hydrophobic surface on ubiquitin characterized by the Ile44-centered patch, including residues Leu8, Ile44, His68, and Val70. Residues surrounding this patch allow for a specific modality of recognition that is peculiar of each domain family, thus explaining how different domains have evolved towards the recognition of a common hydrophobic contact site.

Neural precursor cell expressed developmentally downregulated protein 8 (NEDD8) is the closest relative to ubiquitin (58% identity and 80% similarity). Both molecules present a similar asymmetric surface distribution of charges with a predominantly acidic face opposed to a hydrophobic one. Notably, the Ile44-centered patch is identical in ubiquitin and NEDD8 molecules [5]. The high degree of conservation in the hydrophobic surfaces provides the explanation of why NEDD8 is usually involved in the recognition by several UBDs with affinities that are similar to those reported for the interactions of such domains with ubiquitin. On the other hand, it also gives rise to the risk of misleading interpretations of the experimental results, since it causes recognition promiscuity that is frequently not confirmed at a physiological level. Therefore, investigating how protein domains can discriminate between NEDD8 and ubiquitin is a challenge that allows tracing the most accurate picture of the biological properties and the cellular functions in which these highly similar proteins are involved.

The NEDD4 binding protein 1 (N4BP1) was originally identified as a target for the proteasomal degradation by the homologous to the E6AP carboxyl terminus (HECT) E3-ligase Nedd4 [6,7]. Indeed, Nedd4 has been shown to post-translationally regulate the intracellular localization of N4BP1 in the nucleolus of primary cells and at promyelocytic leukemia nuclear bodies. The interaction with the HECT ligase Itch, on the contrary, does not seem to be followed by N4BP1 ubiquitination, but rather N4BP1 counteracts the activity of the Itch E3-ligase by directly competing with Itch substrates, such as c-Jun and the members of the p53 family, p63 and p73, thus suggesting a role as promoter of tumor progression [7]. Accordingly, by interacting with the de-ubiquitinating enzyme Cezanne, N4BP1 acts also as a potent negative regulator of nuclear factor-kappa B (NF-κB) signaling in neuroblastoma cells, a role that points to N4BP1 as a candidate target for immunotherapy [8].

By screening protein arrays with the human ubiquitin precursor UbC, Fenner and collaborators identified novel polyubiquitin binding proteins potentially regulating the NF-κB activity. Among them, the region of N4BP1, including amino acids (aa) 209–896, was identified in the screening, and the interaction was experimentally confirmed [9]. By panning a human brain phage-displayed cDNA library using ubiquitin as bait, we identified a shorter fragment of N4BP1 (aa 813–896) that shows ubiquitin binding features, and we further reduced the functional region to the last 50 amino acids of human N4BP1 (aa847–896) by means of biochemical approaches. Based on the mutational analysis, we showed that the interaction between N4BP1 and ubiquitin involves the Ile44-hydrophobic patch [10,11], in analogy with what has been observed in the majority of the UBDs characterized so far.

We present here the structural model of the novel ubiquitin-binding domain identified in N4BP1, both alone and in complex with ubiquitin. Structural analysis performed by nuclear magnetic resonance (NMR) spectroscopy revealed a three alpha-helix bundle domain. Using biochemical assays, we demonstrate that the interacting surface involves the hydrophobic patch in ubiquitin and a key motif in N4BP1 resembling the Phe-Pro (FP) motif typified in the coupling of ubiquitin conjugation to ER degradation (CUE) domain family. Nevertheless, the structural analysis shows that key differences in the ubiquitin-binding domain of N4BP1 do not support the inclusion of this novel domain either in the CUE or in other three-helix bundle domain families, such as ubiquitin-associated domains (UBA). Conversely, the UBD of N4BP1 shares common features with the cullin binding domain associating with NEDD8 (CUBAN), recently identified in the evolutionary related KHNYN protein [10,11]. Based on this feature, we dubbed the UBD domain of N4BP1 as CoCUN domain, for *Cousin of CUBAN*. The observed similarity, both at the primary sequence and the structural level, supports the identification of a novel domain family having the CUBAN and the CoCUN domains as unique members. Finally, we show that both domains are poly-ubiquitinated when expressed in cells and that a functional ubiquitin/NEDD8 binding property is required for the domain in order to be post-translationally modified.

## 2. Results

### 2.1. A Novel Ubiquitin-Binding Domain at the C-Terminal End of Human N4BP1

**Figure 1:**
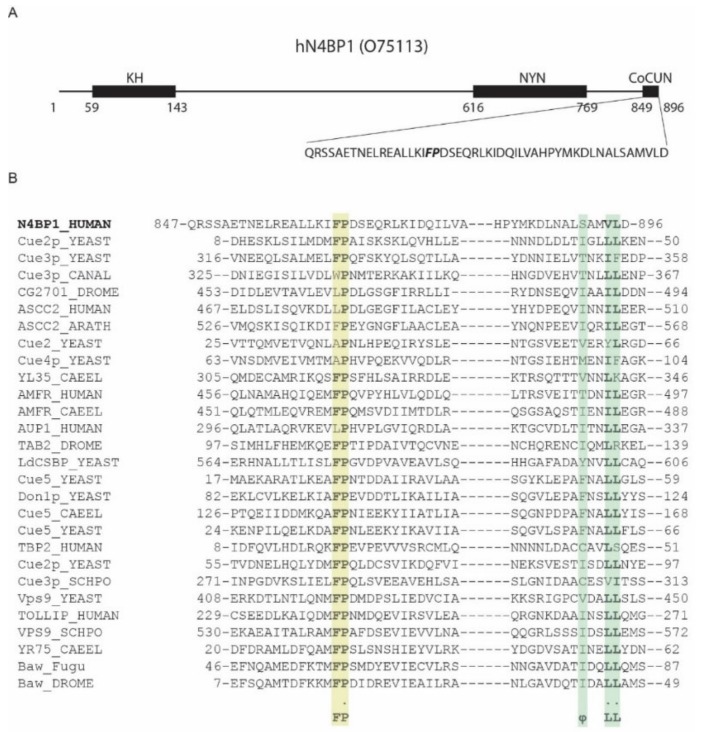
The ubiquitin-binding domain of NEDD4 binding protein 1 (N4BP1) resembles a ubiquitin conjugation to ER degradation (CUE) domain. (**A**) Domain organization of human N4BP1 (UniProt code O75113). The sequence of the carboxyl-terminal end of N4BP1 identified by phage display and spanning residues 813–896 is shown. (**B**) Comparative sequence analysis of the ubiquitin binding domains (UBD) of N4BP1 human with CUE domain containing proteins, performed with SMART search using as query sequence the amino acid region shown in (**A**). Colored backgrounds highlight the phenylalanine-proline (FP) pair with the invariant Proline (yellow) and the C-terminal hydrophobic motif (green).

Fenner and collaborators recently identified N4BP1 as a potential polyubiquitin chains binding protein. A previous analysis performed with the phage display approach enabled us to identify the region spanning residues 813–896 in N4BP1 as the one showing ubiquitin binding features [10]. The bioinformatics analysis of this region, performed with Simple Modular Architecture Research Tool (SMART) database, reported the presence of a CUE domain in the region including residues 847–896 (Figure 1A), whose primary sequence, however, is too divergent from known CUE domain sequences to be reliably considered a member of this group (Figure 1B) [10]. The CUE domains are structurally closely related to the UBA (ubiquitin-associated domains) [2,12]. Indeed, they share the three-helix bundle structural model and both recognize the Ile44 patch of ubiquitin via conserved hydrophobic residues mapping at the C-terminus of the helix-1. In particular, two main features characterize CUE domains—a phenylalanine-proline (FP) sequence and a φxx(I/L/V)L motif, where φ is a large hydrophobic residue [13]. The first hydrophobic motif mapping at the end of helix-1 is essential for ubiquitin binding, and indeed it directly contacts Ile44, His68, Leu69, and Val70 in ubiquitin. As with the FP motif, the C-terminal non-polar φxx(I/L/V)L motif also binds the hydrophobic path surrounding Ile-44 of ubiquitin, and it is involved in domain stability [14,15].

**Figure 2:**
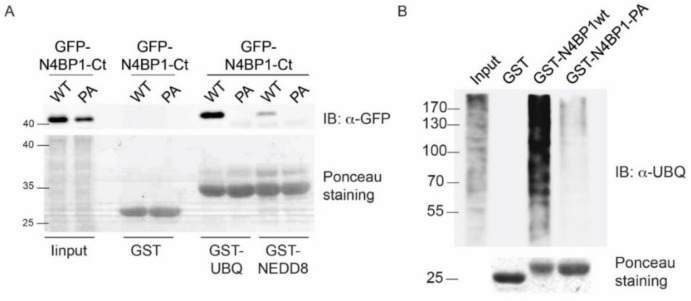
A Pro-Phe motif in N4BP1 is involved in the interaction with ubiquitin and ubiquitinated proteins. (**A**) The amino acid fragment spanning residues 813–896 of N4BP1 and the Pro866Ala mutant (PA) were expressed as GFP fusions in HEK293 cells. Twenty hours post-transfection, cells were harvested, and lysates were incubated with glutathione S-transferase (GST)-ubiquitin, GST-NEDD8, or GST alone. Samples were analyzed by sodium dodecyl sulfate polyacrylamide gel electrophoresis (SDS-PAGE) with anti-GFP antibody. (**B**) Cell extracts from HEK293 cells were incubated with the GST fusions of N4BP1 (813–896aa), N4BP1-PA, or with the GST alone. Samples were analyzed by SDS-PAGE with ubiquitin antibody. Ponceau staining confirmed that equal amounts of purified proteins were loaded.

Interestingly, the minimal ubiquitin-binding region in N4BP1, including residues 847–896, shares 40% identity with the CUBAN domain, recently identified in the evolutionary related KHNYN protein [10]. The CUBAN has been shown to bind NEDD8 with an affinity that is higher than the value measured for monomeric ubiquitin [10]. In addition to interacting with neddylated cullins, due to the direct recognition of the NEDD8 molecule, the CUBAN domain binds ubiquitin chains and poly-ubiquitinated proteins. This dual capability is due to the presence of two binding sites that are in close proximity, as evidenced by the observation that the interaction of the CUBAN domain with ubiquitin chains competes out NEDD8 binding [10,11]. In order to evaluate the functional relevance of the FP motif in N4BP1, we inserted the mutation Pro866Ala (PA mutant) in the amino acid fragment spanning residues 813–896 of N4BP1, and we compared the binding specificities of the two constructs by pull-down assay. GFP-N4BP1 and the PA mutant were independently expressed in HEK293 cells, and the extracts were incubated with beads coated with glutathione S-transferase (GST), GST-ubiquitin, or GST-NEDD8 (Figure 2A). As shown, GFP-N4BP1, but not the PA mutant, was efficiently recovered by ubiquitin. Moreover, in keeping with our previous reports [10], N4BP1 clearly bound ubiquitin, while only a faint signal could be detected in the GST-NEDD8 pull-down. Consistent with the previous result, the GST fusion of N4BP1 bound ubiquitinated proteins from a HEK293 cell extract, and the interaction was abrogated by the Pro866Ala mutation (Figure 2B). These results demonstrate that the FP motif of N4BP1 acts similarly to the corresponding key motif in the CUE domains. On the other hand, sequence similarities indicate that this domain is structurally similar to the CUBAN despite being significantly different in the binding preferences. The overall divergence highlighted by the SMART search prompt us to speculate that the ubiquitin binding domain of N4BP1 has acquired functional characteristics that are common to the CUE domain family despite not being evolutionary related. Given this independent evolution from CUE domains and, above all, the evolutionary relationship with CUBAN, we dubbed the region spanning the C-terminal 50 residues of N4BP1 the CoCUN domain, for *Cousin of CUBAN*.

### 2.2. Nuclear Magnetic Resonance Spectroscopic Study of N4BP1 Ubiquitin Binding Domain

**Figure 3:**
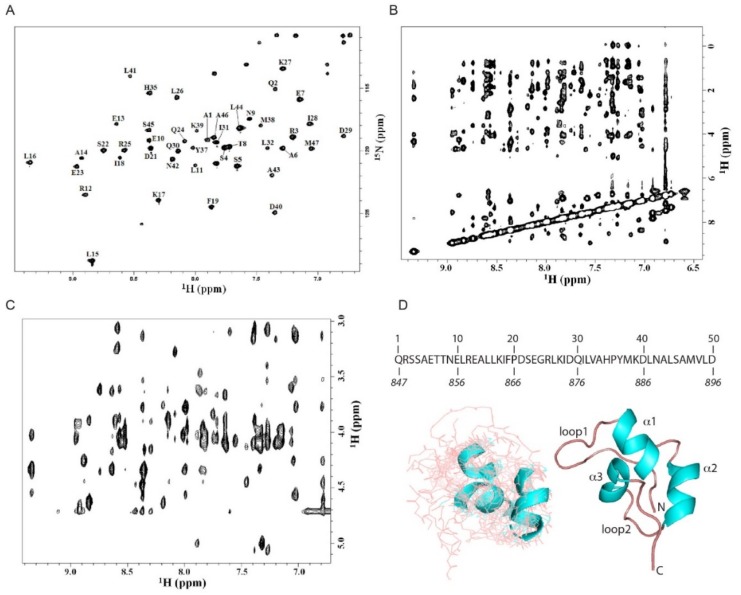
Structural model of the ubiquitin binding domains (UBD) domain of N4BP1. (**A**) ^1^H-^15^N HSQC NMR spectrum of N4BP1 obtained as reported in Materials and Methods. The sequence of the structured region is shown with the relative numbering herein adopted. The numbering of residues referred to the full-length protein is shown in italics at the bottom. (**B**) Amide proton region of the ^1^H-^1^H NOESY spectrum of N4BP1. (**C**) Finger print region of the ^1^H-^1^H NOESY spectrum of N4BP1. (**D**) (Left) Cluster of the top 10 structures (out of 200) obtained by simulated annealing from NMR data of N4BP1. (Right) Cartoon representation showing the secondary structure of N4BP1.

To gain a more accurate description of the CoCUN domain of N4BP1, the NMR spectroscopy study of the C-terminal end (residues 847–896) using the two-dimensional (2D) and the three-dimensional (3D) NMR methodology was performed, leading to the assignment of 47 out of 50 amino acids in the ^15^N labeled protein. The ^15^N heteronuclear single quantum coherence (HSQC) spectrum is reported in Figure 3A together with the domain primary sequence. The spectral dispersion was sufficient to achieve the assignment of the resonances obtained by 3D ^15^N-HSQC-TOCSY (total correlation spectroscopy) and 3D ^15^N-HSQC-NOESY (nuclear Overhauser spectroscopy) spectra. The cross-peaks were labeled, and the assignments were further confirmed by ^1^H TOCSY and ^1^H NOESY spectra obtained with different mixing times as reported in Materials and Methods (Figure 3B). As shown, the nuclear Overhauser effects (NOEs) number in the fingerprint region of the ^1^H NOESY spectrum of N4BP1 was not as large as expected for a fully structured protein of this size (Figure 3C), indicating the presence of an extended random coil region in the secondary structure. The analysis of the ^15^N chemical shifts of N4BP1 performed with the algorithms of Wishart and Sykes [16] led to the identification of the secondary structure elements of CoCUN, consisting of three regions (α1, α2, and α3) whose chemical shift deviations are typical of a helix conformation. Only a single value characteristic of a beta secondary structure without any persistence of this chemical shift deviation was identified, thus excluding the presence of secondary structures other than α-helices. The complete assignment of resonances was performed and is reported in Table 1.

Based on the NOEs observed, the structural model of the CoCUN domain was calculated using the conventional software package XPLOR [17]. Simulated annealing protocol in vacuo [18], inter-protons distances and torsion angles (φ and ψ) extracted from NMR data (NOEs and chemical shift index) were used as restraints during the molecular dynamics simulations. Beyond the three helices, a large part of the protein appeared in a random coil conformation, indicating a pronounced structural flexibility, which was also confirmed by the limited number of NOEs that were detected and the absence of interhelical NOEs. Figure 3D reports the average of a cluster of 10 structures chosen by the minimum energy level out of the 200 generated by XPLOR. The domain conformation scheme recalls the structural features of the largest group of ubiquitin-binding domains, represented by the three-helix bundle fold [2]. Notably, the long loop connecting helices α1 and α2 presented a high level of flexibility, a feature resembling the extended loop1 of the evolutionary related CUBAN domain [10,11].

### 2.3. Conformational Studies of the CoCUN Domain in Complex with Either Ubiquitin or NEDD8 Performed by Circular Dichroism

**Figure 4:**
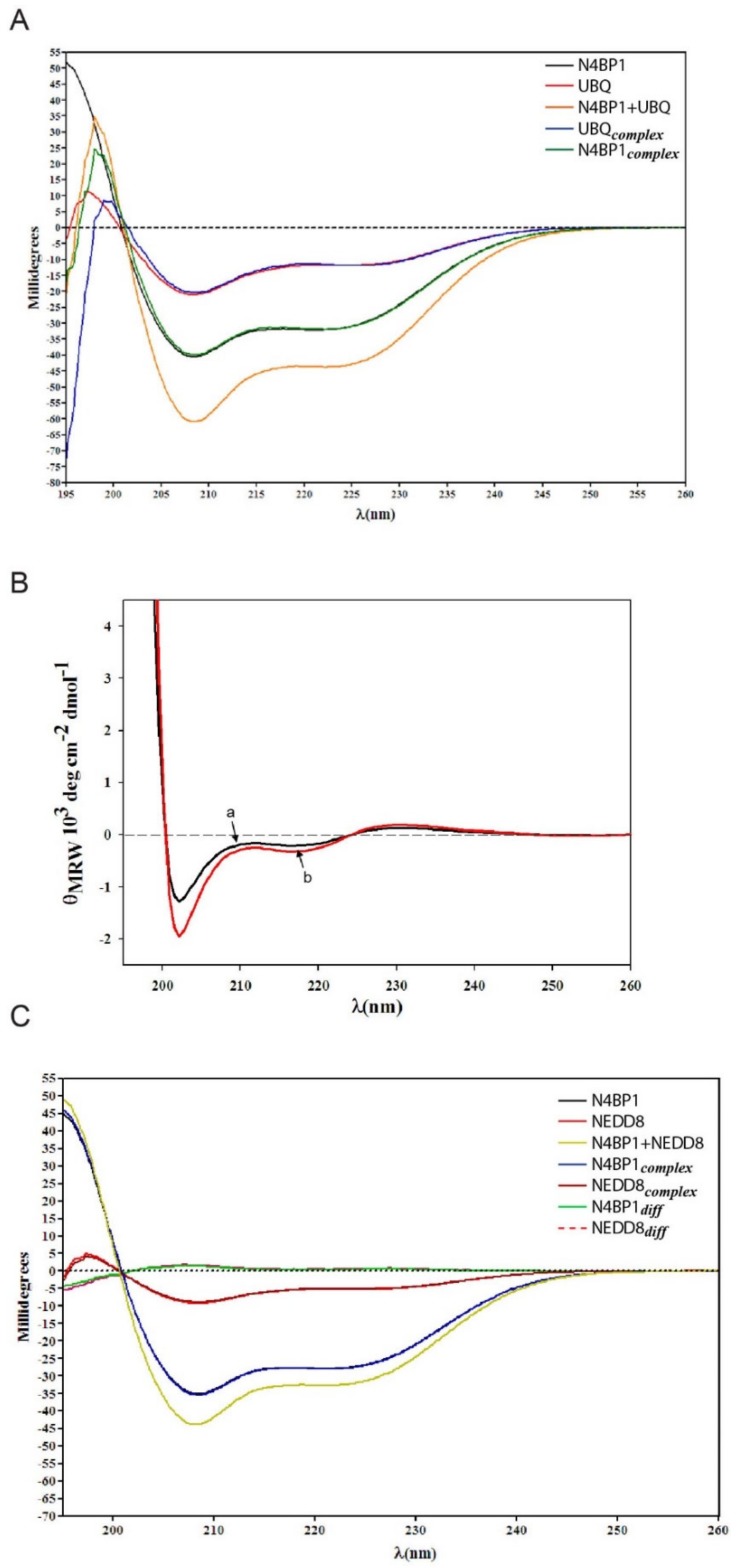
Conformational studies of N4BP1 in complex with either ubiquitin or NEDD8, performed by circular dichroism (CD). (**A**) CD spectra of N4BP1 and ubiquitin alone expressed as rotatory power are shown in black and red traces, respectively. The orange trace reports the CD spectrum of the 1:1 molar mix of N4BP1 and ubiquitin at the concentration of 30 µM. The green trace (N4BP1_complex_) reports the CD spectrum of N4BP1 in the complex obtained after subtracting point-by-point from the CD of the complex (orange) and the CD spectrum of ubiquitin alone (red). Similarly, the blue trace reports the CD spectrum of ubiquitin in the complex obtained after subtraction of the CD spectrum of N4BP1 alone (black). (**B**) The differences shown in (**A**) are amplified and here reported as mean residue ellipticity. The black trace (a) represents the amplification of the point-by-point difference in the CD spectra between ubiquitin in complex with N4BP1 and ubiquitin alone. The red trace (b) represents the amplification of the point-by-point difference in the CD spectra between N4BP1 in complex with ubiquitin and N4BP1 alone. (**C**) CD spectra of N4BP1 and NEDD8 alone are shown in black and red traces, respectively. The yellow trace reports the CD spectrum of the 1:1 molar mix of N4BP1 and NEDD8 at the concentration of 30 µM. The blue and the brown traces report the CD spectra, respectively, of N4BP1 after subtracting the spectrum of NEDD8 from the spectrum of the mixture and of NEDD8 after subtracting the spectrum of N4BP1 from the spectrum of the mixture. These differences are reported in green (N4BP1_diff_) and dashed red (NEDD8_diff_) traces, respectively.

To gain more information on changes in the secondary structure of the N4BP1 UBD upon binding, the CD spectra were obtained in the region from 195 to 260nm, as reported in Figure 4A. By applying different optimization and deconvolution techniques, we obtained an estimate of secondary structure content that resulted to be about 46% α-helix and 3% β-sheet (or β-turn) when using the K2D3 method [19], a value confirmed also by using DICHROWEB [20] with a deconvolution optimized by the fitting routine CONTIN (~45% α-helix content) [21]. In both cases, the estimate was in agreement with the results obtained by NMR. Indeed, the helix content found in the NMR structural model and deduced by the chemical shift index, involves the 47% of the sequence, particularly the tracts 6–12, 23–32 and 37–45. Finally, the NMR result is also supported by the Agadir prediction (http://agadir.crg.es) [22] which gives an estimate of 45% total helix content, corresponding to the tracts 6–18, 23–31 and 43–47. 

CD spectroscopy is widely used for the extraction of elements of secondary structure of polypeptides. This practice uses a fitting that often does not give very good results depending on the database applied. On the contrary, CD remains an optical physical chemistry measure, which is a sensitive monitor of secondary structure changes of proteins and of their interactions [23,24,25]. We then used the CD spectroscopy to report changes in the ellipticity related to the secondary structure elements of the different proteins in the CoCUN/ubiquitin complex and we compared them with the CD spectra obtained for the CoCUN/NEDD8 mix. Figure 4A shows the CD spectra of CoCUN and ubiquitin, both alone and in the presence of an equimolar amount of the binding partner (experimental details are reported in Materials and methods). We also measured the spectral differences between each component in the mixture and the single, isolated component. As shown, the point by point differences between the CD spectrum of N4BP1 and ubiquitin in the mixture and their CD spectra when measured alone, reveal that small conformational changes, although different in shape and intensity, occurred in the CD spectra. The small values found in both dichroic profiles are in line with partial adjustments of the conformation upon binding between the two proteins, thus indicating that a mutual perturbation occurred due to an intermolecular interaction. The observed tiny differences were amplified and reported in Figure 4B. A similar experiment, performed with NEDD8 in place of ubiquitin, showed that, within the experimental error, traces in the differences of the CD spectra are close to zero for both proteins (Figure 4C), thus confirming that no interaction occurred between the CoCUN domain and NEDD8, in agreement with previous observations.

### 2.4. ^15^N The HSQC NMR Investigation of the CoCUN/Ubiquitin Complex by Chemical Shift Perturbation

**Figure 5:**
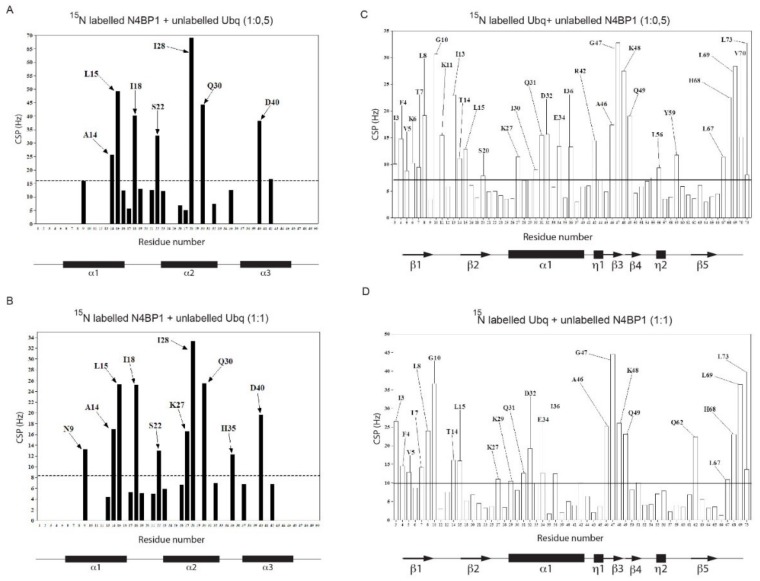
(**A**,**B**) Chemical shift perturbation spectra of ^15^N labeled N4BP1 in the presence of ubiquitin respectively at 1:0.5 and 1:1 molar ratios. (**C**,**D**) Chemical shift perturbation spectra of ^15^N labeled ubiquitin in the presence of N4BP1 at 1:0.5 and 1:1 molar ratios, respectively.

We then investigated the CoCUN/ubiquitin complex by monitoring the chemical shift changes(s) of ^15^N labeled CoCUN domain upon addition of unlabeled ubiquitin at different molar ratios. The typical chemical shift perturbations (CSPs) plot is reported in Figure 5A (1:0.5) and Figure 5B (1:1). As shown, residues that were primarily involved in the interaction between ^15^N labeled CoCUN and ubiquitin were A14, L15, I18, S22, I28, Q30, and D40 at both molar ratios. Residues N9, K27, and H35 were only perturbed at the 1:1 molar ratio. The complementary experiment performed using ^15^N labeled ubiquitin and the unlabeled CoCUN domain showed that a large number of residues were perturbed following the addition of the unlabeled protein at both 1:0.5 (Figure 5C) and 1:1 molar ratios (Figure 5D) of ^15^N-labeled ubiquitin/unlabeled CoCUN. Fixing a threshold of 1σ (indicated by a line), a view of the residues involved in the interaction could be highlighted. As shown, the hydrophobic residues mapping in the β-sheet region of ubiquitin were the main determinants of the interaction, followed by a large number of polar and neutral residues that were also involved, thus indicating a complex recognition mechanism between the two proteins upon the early hydrophobic recognition step. In order to determine the regions involved in the interaction, CSPs detected by both experiments were analyzed by the HADDOCK procedure as reported in Materials and Methods [26].

**Figure 6:**
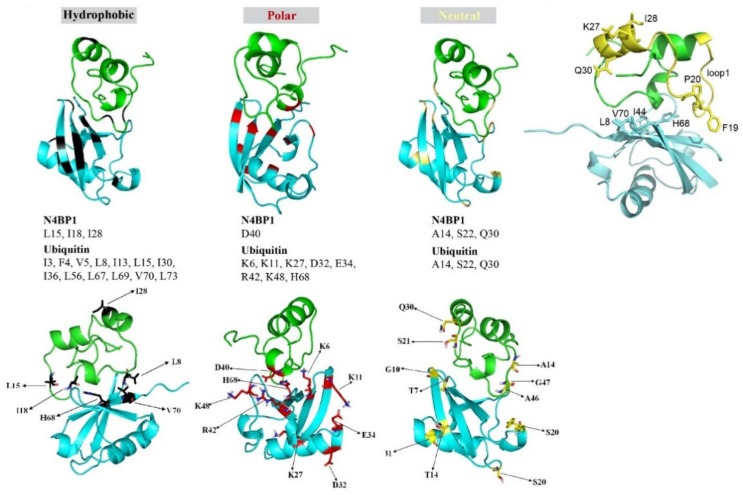
Conformational model of the N4BP1/ubiquitin complex obtained by combining the structural model of N4BP1, the structure of ubiquitin (BMRB 6457 and PDB 1D3Z), and the reported chemical shift perturbation (CSP) values (upper panels). Colors indicate the nature of the residues influenced by the interaction. Black: hydrophobic residues, red: polar residues; yellow: neutral residues. Sticks represent side chains of the amino acids whose ^15^N resonances are perturbed upon interaction with ubiquitin (lower panels). The distal residues K27, I28, and Q30 in helix-2 show significantly perturbed ^15^N resonances upon interaction of N4BP1 with ubiquitin. Structural model showing residues in helix-2 (K27, I28, and Q30) that are perturbed in the N4BP1/ubiquitin complex. The side chains of the FP pair in the loop1 and the K27, I28, and Q30 residues in helix-2 are shown in sticks (see details in the text).

The model reported in Figure 6A (upper panels) shows that helix-1 and loop-1 in CoCUN were both involved in ubiquitin binding. On the other hand, ubiquitin recognized the UBD domain of N4BP1 by means of residues mapping in the β-sheet secondary structure. Particularly, a detailed analysis of the hydrophobic, the polar, and the neutral residues, whose chemical shifts were perturbed by the interaction, revealed that Leu8, His68, and Val70 in ubiquitin were all involved in the interaction with the CoCUN domain. These residues together with Ile44 define the well-known interacting surface of the Ile44-patch, which is central to the majority of UBD/ubiquitin interactions characterized to date. Even though Ile44 lacks a measurable chemical shift perturbation (CSP), the mutation Ile44Ala abrogates the interaction, thus definitely confirming that the hydrophobic patch of ubiquitin is the primary contact site. On the other hand, residues V48 and L49 at the end of helix-3 did not show any perturbation. It is important to remark that, beyond the hydrophobic contacts, many polar and neutral residues are also involved, thus confirming the steering role of electrostatic and/or dipolar interactions during the protein–protein recognition. Further investigation will allow us to better refine the binding requirements of this interaction.

Figure 6B shows the relevant CSP perturbations of K27 (at 1:1 molar ratio), I28, and Q30 (both at 1:0.5 and 1:1) in N4BP1, which were located quite far from the interacting surface (see also Figure 5A,B) and therefore should not have been affected by the interaction. A detailed observation of the complex structural model may lead to an intriguing interpretation of this result. Indeed, given that CSPs are due to perturbations of the ^15^N backbone chain caused by conformational changes of either the side chain or the secondary structure tract itself, the CSP observed for these residues could be considered as an indicator of magnetic environment perturbations of the ^15^N of the amide bond due to a secondary structure perturbation in that tract. Based on this premise, a possible explanation for the relevant perturbations of K27, I28, and Q30 upon binding with ubiquitin could be that the tract of helix-2 containing these residues was subjected to helical geometry distorting forces generated by the pulling down effects of the two flanking loops 1 and 2 toward the ubiquitin-interacting surface.

### 2.5. The CoCUN Domain Binds Monoubiquitin, Albeit with Low Affinity

**Figure 7:**
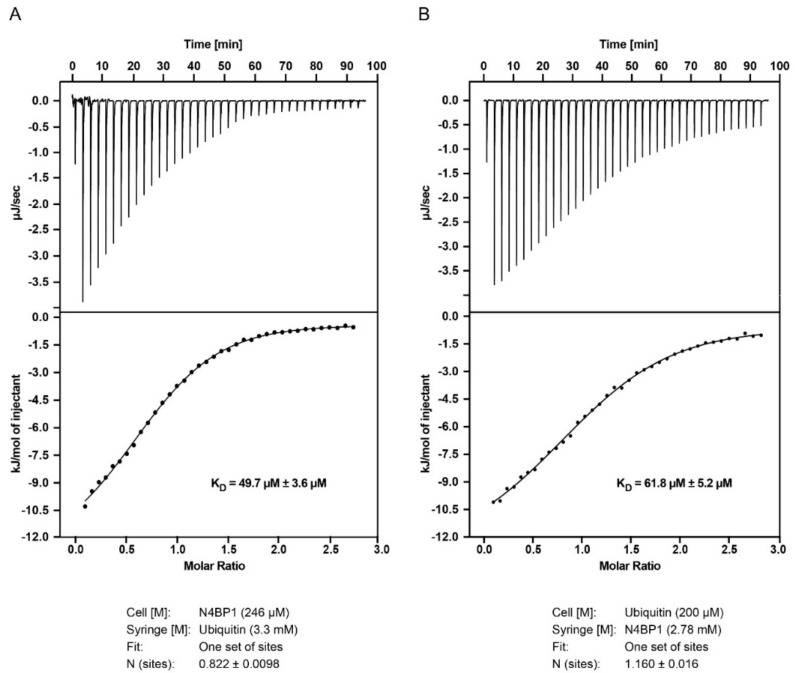
Isothermal titration calorimetry (ITC) experiments with N4BP1 and ubiquitin. (**A**). Titration of 3.3 mM ubiquitin to 246 µM N4BP1 (**B**) Titration of 2.78 mM N4BP1 to 200 µM ubiquitin. The data for the two titrations show similar dissociation constants (K_D_).

Ubiquitin-binding domains directly bind monoubiquitin with variable affinities, going from 10 µM to 500 µM. In order to measure the ubiquitin binding affinity of CoCUN domain, we performed isothermal titration calorimetry (ITC) with the purified N4BP1 polypeptide (aa 847–896) (Figure 7A). The dissociation constant and the enthalpy of ubiquitin binding to the CoCUN domain were K_D_ = 49.7 ± 3.6 µM and ∆H = −12.6 ± 0.3 kJ/mol, respectively (Figure 7A). We also measured the interaction by inverting samples, thus titrating the CoCUN domain to ubiquitin. The results reported a K_D_ = 61.8 ± 5.2 µM and ∆H = −13.0 ± 0.4 kJ/mol, in agreement with the previous measurement (Figure 7B). Compared to other UBDs, the binding of CoCUN domain in complex with monoubiquitin appeared to be in the range of the weakest CUE/ubiquitin interactions [13].

### 2.6. Differences and Similarities among CoCUN, CUE, and CUBAN

**Figure 8:**
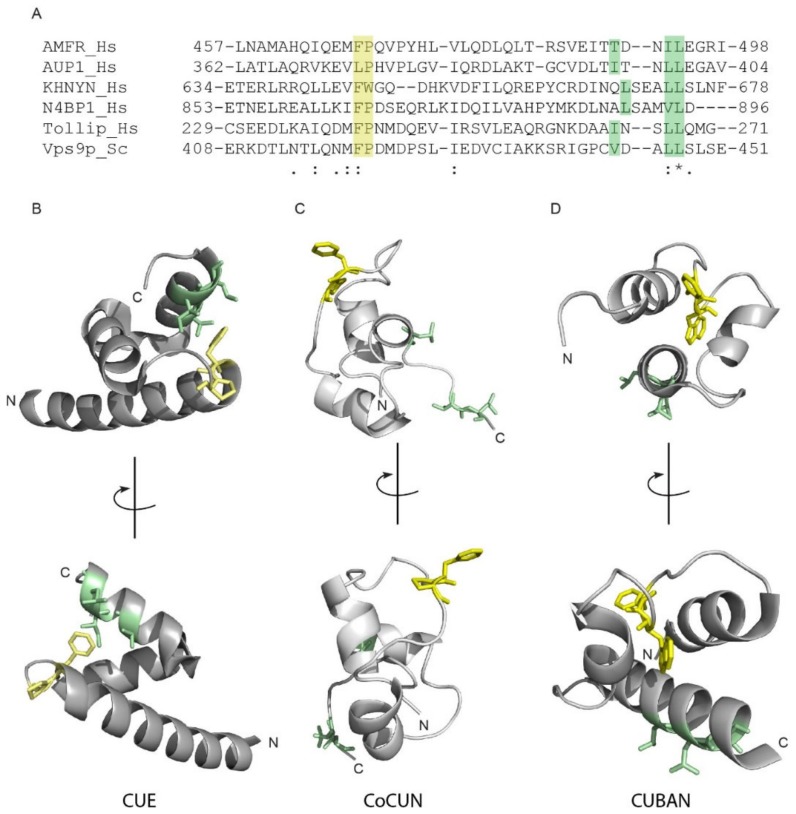
(**A**) Multiple sequence alignment between KHNYN, N4BP1, and members of the CUE domain protein family. Amino acid ranges: hAMFR (aa 457–498), hAUP1 (aa 362–404), hKHNYN (aa 634–678), hN4BP1 (aa 853–896), hTOLLIP (aa 229–271), and ScVps9p (aa 408–451). The FP/FW and the di-leucine motifs are highlighted in grey boxes. Consensus symbols in the alignment: ‘.’ indicates conservation between groups of weakly similar properties; ‘:’ indicates conservation between groups of strongly similar properties; ‘*’ indicates positions which have a single, fully conserved residue. Coloured boxes show the FP pair (yellow) and the φxx(I/L/V)L motif (brown). (**B**–**D**) Cartoon representations of the CUE domain of AMFR (B, PdB 2EJS), the UBD of N4BP1 (**C**) and the CUBAN domain of KHNYN (**D**, PdB 2N7K). The FP/FW and the C-terminal hydrophobic motifs of N4BP1, AMFR, and KHNYN are shown in sticks colored as in (**A**).

The CUE domains are closely related to UBA [2,12]. Indeed, they share the three-helix bundle structure and both interact with the Ile44 patch of ubiquitin via conserved hydrophobic residues at the C-terminus of the helices α1 and α3. As previously described, the Ile44 patch contact sites in CUE domains are represented by the FP pair and the hydrophobic sequence φxx(I/L/V)L [14,15]. Figure 7A shows the alignment of the CoCUN domain of N4BP1 with the evolutionary related CUBAN domain in hKHNYN, the CUE domains of human AMFR, AUP1, and Tollip, and the yeast CUE domain of Vps9p. As shown, the sequence φxx(I/L/V)L is represented by the motif LSxx(L/V)L, with a Ser following the hydrophobic leucine in both proteins. Moreover, an FW pair substitutes the FP motif in KHNYN. We therefore inspected the spatial distribution of Phe-Pro side chains with respect to the hydrophobic cluster in CoCUN, CUBAN, and the typical CUE of hAMFR, respectively (Figure 8B–D).

**Figure 9:**
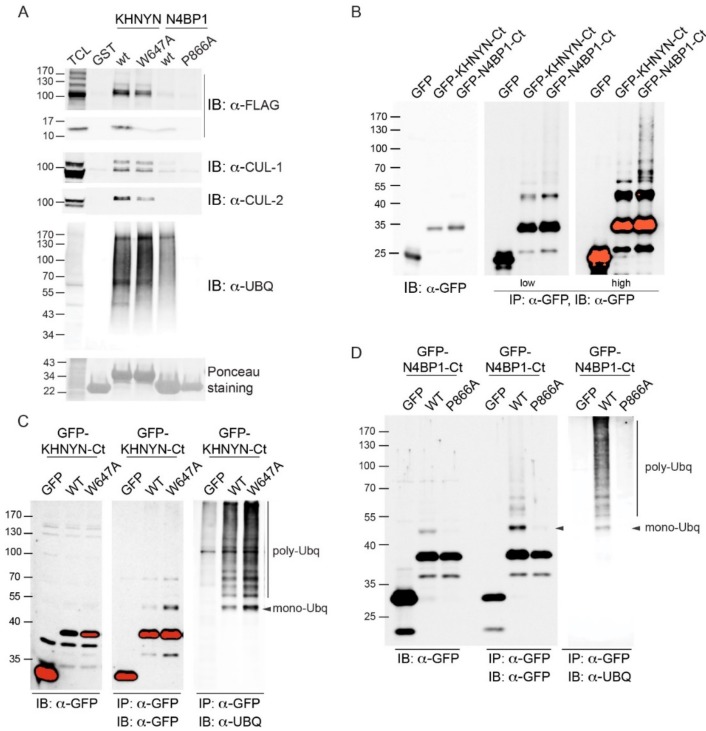
(**A**) The GST fusions of KHNYN (aa 598–678) and N4BP1 (847–896), respectively including the wild-type carboxyl-terminal ends and the corresponding mutants in the FW (W647A) and the FP (P866A) motifs were analyzed by pull-down from a cell extract from T-REx-Flag-NEDD8 cells expressing flag-tagged NEDD8 after incubation with 100 nM doxycycline for 18 h. After washing, the beads were analyzed by SDS-PAGE with anti-Flag, anti-CUL1, anti-CUL2, and anti-ubiquitin. (**B**) The C-terminal regions of KHNYN and N4BP1 mediate the intra-molecular ubiquitination of the domains. The C-terminal end of KHNYN (aa 598–678) and N4BP1 (aa 813–896) were expressed as GFP fusions in HeLa cells and immunoprecipitated with anti-GFP antibody. Beads were washed and analyzed by SDS-PAGE with anti-GFP antibody. Two different acquisition times are shown. (**C**) The mutation W647A in KHNYN does not affect the ubiquitination of the C-terminal end. The KHNYN C-terminal end, both wild type and mutated in the FW motif (W647A), was transfected in HeLa cells and analyzed as described in (**B**). Red bands represent western blotting signals that reached the saturation point. The immunoprecipitates were analyzed by SDS-PAGE with anti-GFP and anti-ubiquitin antibodies. (**D**) The mutation P866A abrogates the polyubiquitination of N4BP1. The N4BP1 C-terminal end, both wild type and mutated in the FP motif (P866A), was transfected in HeLa cells and analyzed as described in (**C**).

**Figure 10:**
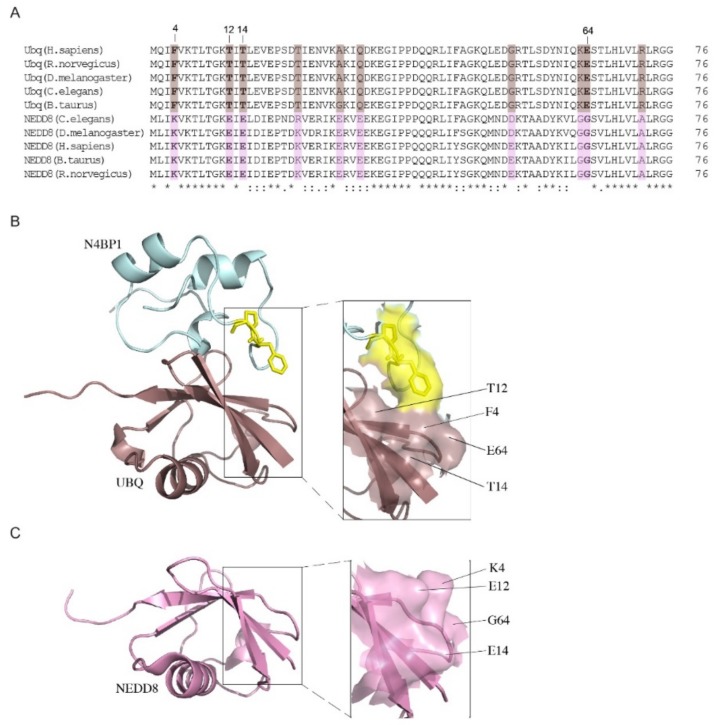
(**A**) Amino acid sequence alignment of ubiquitin and NEDD8 orthologs performed with Clustal Omega (https://www.ebi.ac.uk/Tools/msa/clustalo). Consensus symbols used in the alignment: ‘.’ indicates conservation between groups of weakly similar properties; ‘:’ indicates conservation between groups of strongly similar properties; ‘*’ indicates fully conserved residue. Conserved/divergent positions are highlighted in colored boxes (brown for ubiquitin, violet for NEDD8). (**B**) Complex between the UBD of N4BP1 and ubiquitin. The conserved/divergent patch in ubiquitin (brown) is represented by F4, T12, T14, and E64 (shown in bold in the sequence alignment). (**C**) The corresponding pattern in NEDD8 (violet) includes residues K4, E12, E14, and G64. The magnification of both patterns is shown. Protein models were obtained with PyMol using the HADDOCK model coordinates.

Differences and similarities were clearly visible; while the FP motif was positioned at the C-terminal end of helix-1 in the CUE domain of AMFR, both the FP and the FW pairs in CoCUN and CUBAN occupied a central position inside the extended loop1. Nevertheless, the Trp647 pointed towards the core of the domain and, together with flanking residues, constituted a sort of pivot that reduced the flexibility of loop1 in CUBAN [10]. On the contrary, the FP motif in CoCUN was highly exposed to the solvent, at least in the absence of the ligand. Given the high flexibility of this region, we expected that its spatial position would be significantly affected by the protein complex. In keeping with this observation, the FP pair was directly involved in the interaction with ubiquitin (see Figure 2 and [10]). We therefore attempted to clarify whether the FW and the FP motifs in CUBAN and N4BP1 carboxyl-terminal domains, respectively, were functionally similar (Figure 9). To this end, we compared by pull-down assay the effect of the mutations Trp648Ala and Pro866Ala in the interaction with endogenous neddylated and poly-ubiquitinated proteins. The GST fusions of KHNYN and N4BP1 C-terminal ends, both wild-type and mutated, were incubated with a cell extract from T-REx Flag-NEDD8 cell line, which expresses low levels of flag-tagged NEDD8, under the control of a tetracycline inducible promoter [10]. As shown in Figure 9A, while the mutation Pro866Ala in N4BP1 (P866A) abrogated the interaction with poly-ubiquitinated proteins, the corresponding KHNYN mutant (W647A) retained the capability to interact with both neddylated CUL1 and CUL2 as well as with ubiquitinated substrates despite showing a significant reduction in the binding efficiency towards monomeric NEDD8. This effect was probably the result of conformational variations in the mutated domain, which mainly affected the interaction with the weaker binding affinity. Therefore, while the FW pair in KHNYN showed structure stabilizing features rather than being involved in protein–protein interactions, the FP motif in N4BP1 directly participated in the interaction of the UBD with ubiquitin.

Finally, it has been shown by others that the ubiquitin binding domains UIM and CUE, apart from mediating the interaction with ubiquitinated partners, are required for intra-molecular ubiquitination within the same protein [13,27,28]. We therefore set out to find if both the CUBAN domain and the UBD of N4BP1 would share similar properties. To this end, we transiently expressed both domains as GFP fusions in HEK293 Phoenix cells, and we analyzed the anti-GFP immunoprecipitates with anti-GFP antibody (Figure 9B). As shown, both domains were subjected to several post-translational modifications compatible with mono- and multiple ubiquitinations. The result indicates that both the CUBAN domain and the UBD identified in N4BP1 mediate not only the interaction with ubiquitinated proteins but also covalent modifications that can be predicted to regulate the functional properties of the proteins themselves [10]. We therefore investigated whether the FP and the FW motifs were also involved in the post-translation modifications of the respective protein domains. To this end, we transfected HEK293 Phoenix cells with plasmids coding for the mutants KHNYN_Ct_-W647A and N4BP1_Ct_-P866A fused to the GFP, and we performed the immunoprecipitation with anti-GFP antibody. The immunoprecipitated samples were analyzed by immunoblotting with the anti-ubiquitin antibody (Figure 9C,D). The results confirmed that both domains were ubiquitinated when transiently transfected in cells. Whilst the mutation in the FW motif of KHNYN did not show any effect on protein ubiquitination, the inactivation of the FP motif in N4BP1 abrogated the post-translational modifications of the C-terminal end (Figure 9D). Taken together, our data clearly demonstrate that, despite the two evolutionary related domains sharing the capability to interact with poly-ubiquitin and mediate the multi-ubiquitination of the proteins themselves, they must substantially differ in the way these recognitions take place.

## 3. Discussion

Sixteen different ubiquitin-binding domains have been identified thus far. They can be grouped in different classes depending primarily on the specific structural features. The vast majority belongs to the group of three-alpha helical bundle domains, all sharing the common features of a similar structural fold and the recognition of the Ile44-centered hydrophobic patch in the ubiquitin molecule. Recently, a novel ubiquitin-binding domain was identified at the C-terminal end of KHNYN (also called KIAA0323) protein. This domain, called CUBAN for cullin binding domain associating with NEDD8, shows a clear preference for monomeric NEDD8 and mediates the interaction of KHNYN with neddylated cullins [10,11]. Although the CUBAN also binds ubiquitin in the multimeric form, it was shown that the recognition of ubiquitin and NEDD8 involves different binding sites, thus supporting the notion that the CUBAN domain has evolutionary gained the capability to discriminate between the two highly related molecules. N4BP1 (KIAA0615), a close relative of KHNYN, also contains a ubiquitin-binding domain at the C-terminal end of its amino acid chain (aa 847–896) that shows a K_D_ for monoubiquitin of about 50 µM with a molar ratio 1:1, as also confirmed by the K_D_ measure of about 61 µM for the binding of ubiquitin to N4BP1 (Figure 7).

The sequence analysis of this novel domain revealed a 40% identity and 47% similarity with CUBAN [10,11]. Moreover, the inspection of the carboxyl-terminal ends of KHNYN and N4BP1 structures, both obtained by NMR, showed, respectively, a moderate and a pronounced flexibility, which makes rigorous and comprehensive comparison between the two conformational models difficult. Nevertheless, the 3D conformation of the N4BP1 UBD domain, here dubbed CoCUN (*Cousin of CUBAN*) domain, is more similar to CUBAN with respect to CUE and UBA domains. Intriguingly, despite the high level of similarity, these domains show significant differences in their binding preferences. The circular dichroism spectroscopy confirmed that the interaction of CoCUN with ubiquitin occurs with slight mutual changes in the dichroic profiles of both molecules in line with the structural stability of the ubiquitin molecule, characterized by β-strands flanked by one another with a network of hydrogen bonds. The same experiment performed with the close relative NEDD8 did not report any variation in the dichroic profiles, thus confirming that N4BP1 does not bind to NEDD8. Although tiny these differences indicate that small changes occurred in both CoCUN and ubiquitin, thus suggesting a mutual conformational change [23,24,25]. On the other hand, it is important to remark that several CSPs observed in ^15^N labeled ubiquitin and in the CoCUN domain are located in regions corresponding to the loops connecting secondary structure elements, indicating that changes in the conformational freedom could be the basis of the CD effects.

The interaction study performed by NMR spectroscopy revealed that a hydrophobic region in N4BP1 contacts the hydrophobic patch located in the β-region of ubiquitin. This observation is in line with the molecular details characterizing other ubiquitin binding domains showing a similar structure, such as UBA and CUE, which, despite there being several different micro-environmental details depending on their local structural characteristics, contact the Ile44-hydrophobic patch of ubiquitin [2]. The residue Ile44 is mostly buried at the intermolecular contact area of ubiquitin, thus giving an explanation of the absence of a measurable CSP of the amide ^15^N of the backbone for this residue in our NMR binding experiments. Nevertheless, as expected, the mutational analysis confirmed a clear involvement of this residue in the interaction with N4BP1 ([10,11]). Taken together, our data definitely demonstrate that the hydrophobic patch of ubiquitin, consisting of Leu8, Ile44, and Val70 side chains flanking His68, represents the primary contact site of the N4BP1 CoCUN domain. Further steering contacts take place in this interaction, involving polar and neutral residues that electrostatically or dipolarly strengthen the recognition and also appear to play a role in the protein–protein selectivity, in agreement with the observation that different UBDs contact the hydrophobic patch of ubiquitin with modalities that show peculiar differences for each binding domain [2].

The analysis of the residues that differ between ubiquitin and NEDD8 but whose identities are conserved between the various NEDD8 orthologs has led to the identification of two distinct patterns constituted by conserved/divergent residues and aligned along opposite faces, which are expected to mediate NEDD8-specific interactions [5] (Figure 10A). One of these clusters includes residues F4, K12, and E64 in ubiquitin and K4, E12, and G64 in NEDD8. The strongly different nature of these patterns suggests that they can deeply influence the protein recognition. The space-filling representation shows the interaction of the FP pair of CoCUN with ubiquitin and highlights the possible role of the molecular surface, including positions 4, 12, and 64 together with the residue in position 14, in the discrimination between the two molecules (Figure 10B). In fact, the inspection of this molecular surface in the CoCUN/ubiquitin complex shows that this cluster is in close proximity with the FP motif, suggesting that, even though not directly involved in the interaction with ubiquitin (as predictable by the lack of CSPs for these residues), this molecular surface could be involved in maintaining the complex stability. Particularly, the electronic cloud of the aromatic ring of F865 may interact by stacking with the aromatic cloud of the ring of F4 of ubiquitin flanked by 12T, 14T, and E64, with the latter being the unique polar group in the cluster. On the other hand, the corresponding pattern in NEDD8 offers a charged surface characterized by the polar residues 4K, 12E, and 14E, while it lacks any aromatic electronic cloud that could interact with the FP pair (Figure 10C). In summary, we suggest that the exposed side chains in this region represent a peculiar distribution of hindered (threonines), aromatic (phenylalanine), polar charges (lysine and glutamic acid), and flexibility sources (glycine), which are able to strongly influence, in different ways, the protein recognition with a determined substrate. Intriguingly, residues 4, 12, 14, and 64 were recently shown to be molecular determinants for distinguishing between ubiquitin and NEDD8 by ubiquitin-specific peptidase 2 (USP2) [29], thus confirming their key role in the discrimination mechanism.

The N4BP1 was reported to localize in the nucleolus where it is subjected to several post-translational modifications, such as sumoylation and ubiquitination [30]. While the latter affects protein localization, the biological role of N4BP1 poly-ubiquitination is still an unexplored field. It has been suggested that N4BP1 participates in nuclear RNA processing processes and that the Nedd4-mediated monoubiquitination of N4BP1 is required for this function [31]. We show here that the CoCUN domain of N4BP1, when transiently expressed in cells, promotes its own poly-ubiquitination and that an FP motif is required for both covalent and non-covalent interactions with ubiquitin. Analogously, we previously showed that full-length KHNYN is polyubiquitinated in cells and that this modification is abrogated following the deletion of the CUBAN domain [10,11]. In addition, we showed here that the CUBAN domain itself is ubiquitinated when transiently expressed in cells. Intriguingly, many proteins containing ubiquitin-binding domains undergo mono-ubiquitination in a process called coupled mono-ubiquitination, which requires the interaction between a functional UBD and an E3-ubiquitin ligase. Due to the presence of the CUBAN domain, which binds to both free and cullin-conjugated NEDD8, KHNYN can recruit distinct multi-subunit E3 ubiquitin ligase complexes in their active form, an interaction that could in turn promote KHNYN ubiquitination. We expect that N4BP1, which does not share this feature, is mostly regulated by the interaction with the HECT-ligase Nedd4, as also suggested by published data [7]. Interestingly, several examples of coupled mono-ubiquitination are found in the components of intracellular trafficking machineries. KHNYN and N4BP1 could therefore represent novel examples of proteins regulated by coupled-monoubiquitination, involving a new class of ubiquitin binding domain whose functions, still far from being exhaustively characterized, seem to be primarily related with RNA metabolism processes [31].

## 4. Materials and Methods

*DNA constructs and site-directed mutagenesis.* The cDNA constructs coding for the C-terminal ends of hN4BP1 (813–896) and hKHNYN (598–678) were previously described. The minimal ubiquitin binding region of N4BP1, including amino acids 847–896, was cloned in pGex2TK vector using primers R2326 (forward) CAAGGATCCGCTCAGAGATCTTCTGCA and R2473 (reverse) CTCTCGAGTCAATCCAACACCATGGCAG. The T-REx flag-NEDD8 cell line was generated as previously described [10]. Site-directed mutagenesis of CUBAN W647A and N4BP1 UBD P866A were performed using the QuikChange^®^ Site-Directed Mutagenesis Kit (Invitrogen, Frederick, MD, USA) according to the manufacturer’s instructions.

*Protein expression and purification.* The C-terminal fragment of hN4BP1 (aa 847–896) was cloned in pGex2T vector, and the recombinant protein was prepared as previously described [32]. The bacterial expression was performed according to the protocol for NMR sample preparation described by Weber and collaborators [33].

*Cell extracts, immunoprecipitation, and in vivo ubiquitination assay.* Whole-cell extracts were prepared from mammalian cells by lysis in 25 mM Tris pH 7.5, 125 mM NaCl, 1% glycerol, 1 mM MgCl_2_, 1 mM orthovanadate, 5 mM NaF, 0.5% NP-40, 0.5% Triton X- 100, complete proteases inhibitor cocktail (Sigma, St Louis, MO, USA), and complete phosphatase inhibitor (Sigma) for 30 min on ice before clarification by centrifugation. To detect protein in cell lysates, protein samples were separated by sodium dodecyl sulfate polyacrylamide gel electrophoresis (SDS-PAGE) and transferred onto nitrocellulose membrane. Proteins were detected by immunoblotting and visualized by treating the blots with enhanced chemiluminescence (ECL) (Millipore, Burlington, MA, USA). For the pull-down experiments, equimolar amounts of GST-fusion proteins corresponding to the selected clones and GST alone linked to glutathione-Sepharose 4B beads were incubated 1.5 h with 1 mg of cell extract. Beads were washed three times in washing buffer (25 mM Tris pH 7.5, 125 mM NaCl, 1% glycerol, 1 mM MgCl_2_, 1 mM orthovanadate, 5 mM NaF, 0.5% NP-40, proteases inhibitor cocktail, and phosphatase inhibitor cocktail), and bound proteins were electrophoresed on polyacrylamide gel, transferred to polyvinylidene difluoride (PVDF) or nitrocellulose membranes, and detected with specific antibodies. For the coimmunoprecipitation assay cells were plated in 100 mm dishes and grown to 60–70% confluence in culture medium supplemented with 10% fetal bovine serum (FBS). Twenty hours post-transfection, cells were solubilized in lysis buffer. An equal amount of each protein lysate was incubated with anti-Flag M2 affinity gel beads for 2 h at 4 °C and then washed three times with washing buffer supplemented with fresh inhibitors. The protein lysates and the immune complexes were analyzed by Western blot analyses with rabbit anti-GFP and anti-ubiquitin antibodies.

*Antibodies and reagents.* Polyclonal anti-flag was from Sigma, anti-tubulin and anti-ubiquitin P4D1 from Santa Cruz Biotechnology (Santa Cruz, CA, USA), anti-NEDD8 was from Abcam (Boston, MA, USA), and antirabbit and anti-mouse peroxidase-conjugated were from Jackson Immunoresearch (West Grove, PA, USA).

*Cell culture.* HEK293 and Flp-In T-REx-293-Flag-NEDD8 were grown on DMEM (Dulbecco’s modified Eagle’s medium; GIBCO, Waltham, MA, USA) supplemented with 10% FBS (GIBCO) and 100 units/mL penicillin and 100 μg/mL streptomycin (Invitrogen, Carlsbad, CA, USA). Stably transfected Flp-In T-REx-293-Flag-NEDD8 cells were generated as in [10]. The cells were stimulated with 1 μg/mL tetracycline overnight to induce expression of the desired fusion proteins.

*Nuclear magnetic resonance spectroscopy.* The experimental conditions were similar to those previously reported [10]. Proton (^1^H) and ^15^N NMR spectra were obtained at 298 K on a Bruker Advance instrument operating at 700.132 MHz. Nuclear magnetic resonance studies were carried out in 95% H_2_O–5% D_2_O. Two-dimensional and 3D experiments (^15^N-HSQC-TOCSY and 3D ^15^N-HSQC-NOESY spectra with mixing times TOCSY from 0.040 to 0.080 s and for NOESY from 0.080 to 0.3 s) [34,35,36,37] were necessary to obtain the spin systems assignments and, subsequently, the sequential assignments. In order to study the intermolecular interactions, the chemical shift perturbation (CSP) method was applied by the ^15^N-HSQC-NMR. Spectra of the ^15^N labeled N4BP1 domain alone and in presence of NEDD8 or ubiquitin at different molar ratios were run [38]. This pulse sequence was modified with gradient water suppression [39]. To monitor the changes in ^15^N labeled amide groups in the different conditions, TOPSPIN 3.1 (Bruker Spectrospin, CH) and NMRView software packages [40] were used for data processing. The analysis of the CSPs was performed as described in references [41,42].

*Interaction of N4BP1 in complex with ubiquitin and NEDD8 performed by circular dichroism CD.* The circular dichroism spectroscopy (CD) measurements were performed on a Jasco 810 spectropolarimeter (Jasco, Tokyo, Japan) using a 0.1 cm path length cell cuvette. The concentration of N4BP1 was 50 μM in H_2_O. The CD spectra were obtained in the far UV spectral region from 195 to 260 nm using a step resolution of 0.2 nm and a speed scan for spectra collection of 20 nm/min. Spectra obtained were the average of four scans at room temperature. The estimate of secondary structure elements content was obtained using the web server for CD data prediction as described in the text. Circular dichroism studies were performed at a protein concentration of 50 µM and by adopting the scheme described below. In the first step, the CD spectra of N4BP1 and ubiquitin alone were obtained and expressed as rotatory power. Then, the CD spectrum of N4BP1 in the presence of an equimolar amount of ubiquitin (or NEDD8 for comparison as reported below) was performed. In order to identify the possible conformational changes due to protein–protein interactions, the CD spectra, expressed as rotator power and obtained with the components alone, were subtracted point-by-point from the CD spectrum of the protein complex. Specifically, from the mixture N4BP1+ubiquitin, the first subtraction (mixture-ubiquitin) allowed the evaluation of the conformational changes induced on N4BP1 in complex with ubiquitin with respect to N4BP1 alone. Subsequently, by subtracting the CD spectrum of N4BP1 alone from the one of the mixture N4BP1-ubiquitin (mixture-N4BP1), we obtained the conformational changes induced on ubiquitin in complex with N4BP1. Finally, in order to better detect the tiny changes in the CD profiles, the traces of the obtained differences transformed in mean residue ellipticity were vertically amplified. An identical procedure was applied to the study of the interaction of N4BP1 upon binding with NEDD8. In all these experiments, great attention was devoted to maintaining the same protein concentration, buffering system, and ionic strength.

*HADDOCK procedure.* The HADDOCK protocol of ambiguous interaction restraints (AIR) [26] was used as follows. Active residues were considered those in both N4BP1 and UBQ with CSP values higher than the average and that were exposed to solvent. Passive residues were defined by the software itself as those mapping near to the interacting region.

*Isothermal titration calorimetry (ITC).* The ITC measurements were performed on a MicroCal PEAQ-ITC (Malvern Panalytical, Malvern, UK) instrument in ITC buffer (20 mM Tris-HCl pH 8.0, 150 mM NaCl, 5% glycerol). First, ubiquitin was titrated from a stock of 3.3 mM to 246 µM of N4BP1 provided in the reaction cell. The reverse experiment was performed by titrating from a stock of 2.78 mM N4BP1 to 200 µM ubiquitin in the cell. For each experiment, we used 38 injections of 1 µL with 150 s spacing at room temperature with 750 rpm stirring speed. Raw data were analyzed with the integrated analysis tool, and heat production was fitted to a one-site binding model. Plots are displayed with the GraphPad Prism 8.1.2 for macOS (GraphPad Software, San Diego, CA, USA).

## Figures and Tables

**Table 1:** Chemical shift assignments of resonances of ^1^H and ^15^N nuclear magnet resonance (NMR) spectra.

	N	HN	Hα	Hβ1,2	Hγ	Hδ
A1	119.11	7.90				
Q2	115.05	7.34	4.35	1.96		
R3	118.85	7.20	4.00	1.68		
S4	119.71	7.76	4.70	3.85		
S5	121.19	7.66	4.15	3.95		
A6	119.78	7.29	4.06	1.48		
E7	115.90	7.15	4.00	_		
T8	119.64	7.72	4.07	4.05	1.20	
N9	117.40	7.56	4.10	2.70		
E10	119.06	8.43	3.97	1.88		
L11	121.01	8.00	4.37	1.70		
R12	123.40	8.91	4.60	1.61		
E13	117.83	8.64	3.80	1.90		
A14	121.51	8.96	4.60	_		
L15	128.80	8.82	4.60	2.04		
L16	120.89	9.30	4.60	_		
K17	123.89	8.31	4.24	1.59		
I18	120.30	8.60	4.13	_	1.00	
F19	124.40	7.80	4.38	3.06		
D21	119.80	8.43	4.38	2.74		
S22	119.90	8.73	_	3.79		
E23	121.33	9.10	4.60	2.08		
Q24	119.20	8.08	4.10	2.07		
R25	119.90	8.59	4.13	1.85		
L26	115.70	8.13	4.30	_		
K27	113.42	7.26	4.06	1.91		
I28	118.24	7.17	3.79	1.45	0.83	0.04
D29	118.87	6.87	4.70	2.93		
Q30	119.40	8.11	3.90	2.00		
I31	119.30	7.83	4.10	_	0.08 (Hγ2)	−0.08
L32	119.75	7.41	3.31	_		
V33	115.36	8.37	4.57	2.90		
H35	115.36	8.37	3.14	3.14		
Y37	119.73	8.01	5.10	3.01		
M38	117.95	7.47	4.10	2.70		
K39	118.38	8.04	4.08	_		
D40	124.90	7.46	4.36	2.66		
L41	113.90	8.55	4.05	_		
N42	120.64	8.20	4.70	2.86		
A43	121.92	7.37	4.67	1.40		
L44	118.16	7.64	4.15	_		
S45	118.32	8.37	4.58	3.97		0.9 (Hδ1,2)
A46	119.77	7.29	4.05	1.40		
M47	119.80	7.05	4.27	2.04	2.52	

## References

[1] Swatek K.N., Komander D. (2016). Ubiquitin modifications. Cell Res..

[2] Hurley J.H., Lee S., Prag G. (2006). Ubiquitin-binding domains. Biochem. J..

[3] Husnjak K., Dikic I. (2012). Ubiquitin-Binding Proteins: Decoders of Ubiquitin-Mediated Cellular Functions. Annu. Rev. Biochem..

[4] Sokratous K., Hadjisavvas A., Diamandis E.P., Kyriacou K. (2014). The role of ubiquitin-binding domains in human pathophysiology. Crit. Rev. Clin. Lab. Sci..

[5] Whitby F.G. (1998). Crystal Structure of the Human Ubiquitin-like Protein NEDD8 and Interactions with Ubiquitin Pathway Enzymes. J. Boil. Chem..

[6] Murillas R., Simms K.S., Hatakeyama S., Weissman A.M., Kuehn M.R. (2002). Identification of developmentally expressed proteins that functionally interact with Nedd4 ubiquitin ligase. J. Biol. Chem..

[7] Oberst A., Malatesta M., Aqeilan R.I., Rossi M., Salomoni P., Murillas R., Sharma P., Kuehn M.R., Oren M., Croce C.M. (2007). The Nedd4-binding partner 1 (N4BP1) protein is an inhibitor of the E3 ligase Itch. Proc. Natl. Acad. Sci..

[8] Spel L., Nieuwenhuis J., Haarsma R., Stickel E., Bleijerveld O.B., Altelaar M., Boelens J.J., Brummelkamp T.R., Nierkens S., Boes M. (2018). Nedd4 Binding Protein 1 (N4BP1) and TNFAIP3 Interacting Protein 1 (TNIP1) control MHC-1 display in neuroblastoma. Cancer Res..

[9] Fenner B.J., Scannell M., Prehn J.H. (2009). Identification of polyubiquitin binding proteins involved in NF-κB signaling using protein arrays. BBA-Proteins Proteom..

[10] Castagnoli L., Mandaliti W., Nepravishta R., Valentini E., Mattioni A., Procopio R., Iannuccelli M., Polo S., Paci M., Cesareni G. (2019). Selectivity of the CUBAN domain in the recognition of ubiquitin and NEDD8. FEBS J..

[11] Santonico E., Nepravishta R., Mandaliti W., Castagnoli L., Cesareni G., Paci M. (2019). CUBAN, a Case Study of Selective Binding: Structural Details of the Discrimination between Ubiquitin and NEDD8. Int. J. Mol. Sci..

[12] Hicke L., Schubert H.L., Hill C.P. (2005). Ubiquitin-binding domains. Nat. Rev. Mol. Cell Boil..

[13] Shih S.C., Prag G., Francis S.A., Sutanto M.A., Hurley J.H., Hicke L. (2003). A ubiquitin-binding motif required for intramolecular monoubiquitylation, the CUE domain. EMBO J..

[14] Prag G., Misra S., Jones E.A., Ghirlando R., Davies B.A., Horazdovsky B.F., Hurley J.H. (2003). Mechanism of Ubiquitin Recognition by the CUE Domain of Vps9p. Cell.

[15] Kang R.S., Daniels C.M., Francis S.A., Shih S.C., Salerno W.J., Hicke L., Radhakrishnan I. (2003). Solution Structure of a CUE-Ubiquitin Complex Reveals a Conserved Mode of Ubiquitin Binding. Cell.

[16] Wishart D.S., Sykes B.D. (1994). The 13C chemical-shift index: A simple method for the identification of protein secondary structure using 13C chemical-shift data. J. Biomol. NMR.

[17] Brünger A.T. (1993). Assessment of phase accuracy by cross validation: the free R value. Methods and applications. Acta Crystallogr..

[18] Omichinski J.G., Pedone P.V. (1997). The solution structure of a specific GAGA factor-DNA complex reveals a modular binding mode. Nat. Struct. Biol..

[19] Louis-Jeune C., Andrade-Navarro M.A., Perez-Iratxeta C., Louis-Jeune C., Andrade-Navarro M.A., Perez-Iratxeta C. (2012). Prediction of protein secondary structure from circular dichroism using theoretically derived spectra. Proteins Struct. Funct. Bioinform..

[20] Lobley A., Whitmore L., Wallace B.A. (2002). DICHROWEB: An interactive website for the analysis of protein secondary structure from circular dichroism spectra. Bioinformatics.

[21] Sreerama N., Woody R.W. (2000). Estimation of Protein Secondary Structure from Circular Dichroism Spectra: Comparison of CONTIN, SELCON, and CDSSTR Methods with an Expanded Reference Set. Anal. Biochem..

[22] Muñoz V., Serrano L. (1996). Local versus nonlocal interactions in protein folding and stability–An experimentalist’s point of view. Fold. Des..

[23] Greenfield N.J. (2004). Circular dichroism analysis for protein-protein interactions. Methods Mol. Biol..

[24] Dodero V.I., Quirolo Z.B., Sequeira M.A. (2011). Biomolecular studies by circular dichroism. Front Biosci (Landmark Ed)..

[25] Tamburro A.M., Lorusso M., Ibris N., Pepe A., Bochicchio B. (2010). Investigating by circular dichroism some amyloidogenic elastin-derived polypeptides. Chirality.

[26] De Vries S.J., Van Dijk M., Bonvin A.M. (2010). The HADDOCK web server for data-driven biomolecular docking. Nat. Protoc..

[27] Polo S., Sigismund S., Faretta M., Guidi M., Capua M.R., Bossi G., Chen H., De Camilli P., Di Fiore P.P. (2002). A single motif responsible for ubiquitin recognition and monoubiquitination in endocytic proteins. Nature.

[28] Haglund K., Stenmark H. (2006). Working out coupled monoubiquitination. Nature.

[29] Shin Y.C., Chen J.H., Chang S.C. (2017). The molecular determinants for distinguishing between ubiquitin and NEDD8 by USP. Sci. Rep..

[30] Sharma P., Murillas R., Zhang H., Kuehn M.R. (2010). N4BP1 is a newly identified nucleolar protein that undergoes SUMO-regulated polyubiquitylation and proteasomal turnover at promyelocytic leukemia nuclear bodies. J. Cell Sci..

[31] Anantharaman V., Aravind L. (2006). The NYN Domains: Novel Predicted RNAses with a PIN Domain-Like Fold. RNA Boil..

[32] Santonico E., Panni S., Falconi M., Castagnoli L., Cesareni G. (2007). Binding to DPF-motif by the POB1 EH domain is responsible for POB1-Eps15 interaction. BMC Biochem..

[33] Weber D.J., Gittis A.G., Mullen G.P., Abeygunawardana C., Lattman E.E., Mildvan A.S. (1992). NMR docking of a substrate into the X-ray structure of staphylococcal nuclease. Proteins Struct. Funct. Bioinform..

[34] Bax A., Davis D.G. (1985). MLEV-17-based two-dimensional homonuclear magnetization transfer spectroscopy. J. Magn. Reson. (1969).

[35] Marion D., Wüthrich K. (1983). Application of phase sensitive two-dimensional correlated spectroscopy (COSY) for measurements of 1H-1H spin-spin coupling constants in proteins. Biochem. Biophys. Res. Commun..

[36] Braunschweiler L., Ernst R.R. (1983). Coherence transfer by isotopic mixing: Application to proton correlation spectroscopy. J. Magn. Reson..

[37] Wüthrich K. (1986). NMR of Proteins and Nucleic Acids.

[38] Bodenhausen G., Ruben D.J. (1980). Natural abundance nitrogen-15 NMR by enhanced heteronuclear spectroscopy. Chem. Phys. Lett..

[39] Nguyen B.D., Meng X., Donovan K.J., Shaka A. (2007). SOGGY: Solvent-optimized double gradient spectroscopy for water suppression. A comparison with some existing techniques. J. Magn. Reson..

[40] Johnson B.A., Blevins R.A. (1994). NMR View: A computer program for the visualization and analysis of NMR data. J. Biomol. NMR.

[41] Zuiderweg E.R.P. (2002). Mapping Protein−Protein Interactions in Solution by NMR Spectroscopy. Biochemistry.

[42] Williamson M.P. (2013). Using chemical shift perturbation to characterise ligand binding. Prog. Nucl. Magn. Reson. Spectrosc..

