# Erratum: Nepravishta, R., et al. CoCUN, a Novel Ubiquitin Binding Domain Identified in N4BP1. *Biomolecules* 2019, *9*, 284

**DOI:** 10.3390/biom9120803

**Published:** 2019-11-28

**Authors:** Ridvan Nepravishta, Federica Ferrentino, Walter Mandaliti, Anna Mattioni, Janine Weber, Simona Polo, Luisa Castagnoli, Gianni Cesareni, Maurizio Paci, Elena Santonico

**Affiliations:** 1 School of Pharmacy East Anglia, University of Norwich, Norwich NR4 7TJ, UK; nprrvn00@uniroma2.it; 2 Department of Biology, University of Tor Vergata, 00133 Rome, Italy; federica.1.ferrentino@kcl.ac.uk (F.F.); Anna.Mattioni@gmail.com (A.M.); Castagnoli@uniroma2.it (L.C.); 3 Department of Chemical Sciences and Technologies, Tor Vergata University, 00133 Rome, Italy; w.mandaliti@alice.it; 4 IFOM, The FIRC Institute for Molecular Oncology, 20139 Milan, Italy; janine.weber@ifom.eu (J.W.); simona.polo@ifom.eu (S.P.); 5 DIPO, Dipartimento di Oncologia ed Emato-oncologia, Università degli Studi di Milano, 20122 Milan, Italy; 6 Fondazione Santa Lucia Istituto di Ricovero e Cura a Carattere Scientifico (IRCCS), 00143 Rome, Italy

The Biomolecules Editorial Offices wishes to make the following erratum to this paper [1].

The affiliations for Dr. Simona Polo and Gianni Cesareni were incorrect in the published paper in *Biomolecules* [1].

Therefore, this information has been changed by the Editorial Office from:

Ridvan Nepravishta ^1,†^, Federica Ferrentino ^2,†^, Walter Mandaliti ^3,†^, Anna Mattioni ^2^, Janine Weber ^4^, Simona Polo ^4^, Luisa Castagnoli ^2^, Gianni Cesareni ^2,5,6,^*, Maurizio Paci ^3,^* and Elena Santonico ^2,^*

^1^ School of Pharmacy East Anglia, University of Norwich, Norwich NR4 7TJ, UK

^2^ Department of Biology, University of Tor Vergata, 00133 Rome, Italy

^3^ Department of Chemical Sciences and Technologies, Tor Vergata University, 00133 Rome, Italy

^4^ IFOM, The FIRC Institute for Molecular Oncology, 20139 Milan, Italy

^5^ DIPO, Dipartimento di Oncologia ed Emato-oncologia, Università degli Studi di Milano, 20122 Milan, Italy

^6^ Fondazione Santa Lucia Istituto di Ricovero e Cura a Carattere Scientifico (IRCCS), 00143 Rome, Italy

* Correspondence: Cesareni@uniroma2.it (G.C.); Paci@uniroma2.it (M.P.); Elena.Santonico@uniroma2.it (E.S.)

^†^ These authors contributed equally to this work.

To:

Ridvan Nepravishta ^1,†^, Federica Ferrentino ^2,†^, Walter Mandaliti ^3,†^, Anna Mattioni ^2^, Janine Weber ^4^, Simona Polo ^4,5^, Luisa Castagnoli ^2^, Gianni Cesareni ^2,6,^*, Maurizio Paci ^3,^* and Elena Santonico ^2,^*

^1^ School of Pharmacy East Anglia, University of Norwich, Norwich NR4 7TJ, UK

^2^ Department of Biology, University of Tor Vergata, 00133 Rome, Italy

^3^ Department of Chemical Sciences and Technologies, Tor Vergata University, 00133 Rome, Italy

^4^ IFOM, The FIRC Institute for Molecular Oncology, 20139 Milan, Italy

^5^ DIPO, Dipartimento di Oncologia ed Emato-oncologia, Università degli Studi di Milano, 20122 Milan, Italy

^6^ Fondazione Santa Lucia Istituto di Ricovero e Cura a Carattere Scientifico (IRCCS), 00143 Rome, Italy

* Correspondence: Cesareni@uniroma2.it (G.C.); Paci@uniroma2.it (M.P.); Elena.Santonico@uniroma2.it (E.S.)

^†^ These authors contributed equally to this work.

We apologize for any inconvenience caused to readers or to the authors by this mistake. The manuscript will be updated, and the original will remain online on the article webpage.
